# Identifying care-home residents in routine healthcare datasets: a diagnostic test accuracy study of five methods

**DOI:** 10.1093/ageing/afy137

**Published:** 2018-07-26

**Authors:** Jennifer K Burton, Charis A Marwick, James Galloway, Christopher Hall, Thomas Nind, Emma L Reynish, Bruce Guthrie

**Affiliations:** 1 Alzheimer Scotland Dementia Research Centre, University of Edinburgh, Edinburgh, UK; 2 Centre for Cognitive Ageing and Cognitive Epidemiology, University of Edinburgh, Edinburgh, UK; 3 Population Health Sciences Division, School of Medicine, University of Dundee, Dundee, UK; 4 Health Informatics Centre, Ninewells Hospital, University of Dundee, Dundee, UK; 5 Dementia and Ageing Research Group, Faculty of Social Science, University of Stirling, Stirling, UK

**Keywords:** care-home, routine data, test accuracy, sensitivity, positive predictive value, older people

## Abstract

**Background:**

there is no established method to identify care-home residents in routine healthcare datasets. Methods matching patient’s addresses to known care-home addresses have been proposed in the UK, but few have been formally evaluated.

**Study design:**

prospective diagnostic test accuracy study.

**Methods:**

four independent samples of 5,000 addresses from Community Health Index (CHI) population registers were sampled for two NHS Scotland Health Boards on 1 April 2017, with one sample of adults aged ≥65 years and one of all residents. To derive the reference standard, all 20,000 addresses were manually adjudicated as ‘care-home address’ or not. The performance of five methods (NHS Scotland assigned CHI Institution Flag, exact address matching, postcode matching, Phonics and Markov) was evaluated compared to the reference standard.

**Results:**

the CHI Institution Flag had a high PPV 97–99% in all four test sets, but poorer sensitivity 55–89%. Exact address matching failed in every case. Postcode matching had higher sensitivity than the CHI flag 78–90%, but worse PPV 77–85%. Area under the receiver operating curve values for Phonics and Markov scores were 0.86–0.95 and 0.93–0.98, respectively. Phonics score with cut-off ≥13 had PPV 92–97% with sensitivity 72–87%. Markov PPVs were 90–95% with sensitivity 69–90% with cut-off ≥29.6.

**Conclusions:**

more complex address matching methods greatly improve identification compared to the existing NHS Scotland flag or postcode matching, although no method achieved both sensitivity and positive predictive value > 95%. Choice of method and cut-offs will be determined by the specific needs of researchers and practitioners.

## Introduction

Research which analyses routinely-collected healthcare data for whole populations has major advantages [1], but relies on being able to accurately classify individuals’ personal characteristics. Variables like age and sex are reliably recorded, but other characteristics are not, including whether individuals reside in care-homes [2, 3]. Care-home residents have been seriously under-represented in research using routine data [4], despite being a vulnerable population with high healthcare use. If we were able to reliably identify the care-home population using routine data, this would allow greater insights into their health and care needs and resource use. New service models could also be evaluated, generating evidence around the effectiveness of interventions which could then be applied in practice. Researchers internationally have used various methods for matching records of patient addresses to addresses of known care-homes. Methods used include postcode matching (sometimes with exclusion of postcodes that include more than one care-home) which inevitably includes residents of nearby houses, other forms of address matching, and large manual validation exercises [5, 6]. These can be time-consuming and complex, and are usually unsuitable for routine use beyond a specific project, particularly by researchers who lack access to identifiable information to verify their findings.

In Scotland, the Community Health Index (CHI) number is the National Health Service (NHS) unique patient identifier and the Master CHI register records the address recorded by the general practice the patient is registered with [7]. Master CHI also records an ‘Institution Flag’ which is applied by NHS Scotland Practitioner Services Division and signals residential and nursing home residency, but the accuracy of this flag has not been evaluated to our knowledge.

The aim of this study was to examine the performance of the CHI Institution Flag and a range of automated address matching tools for identifying care-home resident addresses using routinely recorded address data.

## Methods

### Study design

The study design is a prospective diagnostic test accuracy study, reported in accordance with the Standards for Reporting of Diagnostic Accuracy Studies (STARD) guidelines [8], with five ‘index test’ methods for automatically allocating whether an address is a care-home address compared to the ‘reference standard’ of independent manual allocation by two researchers.

### Population

The population studied was four random samples of addresses from the NHS Scotland Master CHI register [7]. Using the method described by Hajian-Tilaki [9], a sample size of 4898 was estimated to be adequate to estimate sensitivity of 85% with a 5% marginal error. The University of Dundee Health Informatics Centre (HIC) [10] randomly sampled four independent cross-sectional samples of 5,000 addresses from the NHS Fife and NHS Tayside Master CHI registers on 1 April 2017. NHS Tayside and Fife are the 5th and 7th largest of 14 regional health boards, respectively, comprising 14.5% of the Scottish population combined [11]. Fife is a geographically larger predominantly rural area, while Tayside includes Dundee City, with a population of 148,710 and areas of high deprivation [12]. Combined, the two areas are representative of the Scottish population.

Two samples were obtained from each health board area, one of adults aged ≥65 years and one of all residents. Records were labelled with an anonymous study identification number, retaining the address fields and CHI Institution Flag only, removing other personal identifiers.

### Reference standard

The reference standard was created independently of index test calculation (the output of the address matching methods). Two researchers each independently assigned each patient address to a binary category of ‘care-home address’ or ‘not care-home address’ using record by record comparison with the Care Inspectorate list of current and previous registered services in Scotland, updated on the 30 March 2017 [13]. A ‘care-home’ was defined as a Care Inspectorate mandatory-registered nursing or residential care facility providing 24-h care for its residents. Only Care Home Services registered for Older People were included. Services providing sheltered housing, supported accommodation and extra-care housing were excluded. The two independent assignments of addresses were compared and any disagreements resolved by discussion to create a ‘gold standard’ binary allocation of each address as a care-home address or not.

### Index tests

The index tests examined were chosen to reflect methods: already available to researchers; tested in other published cohorts or using innovative approaches to manage free-text data.

We applied five methods to identify CHI register addresses as a care-home address or not. The methods were as follows:

- The CHI Institution Flag: field in the Master CHI register identifies whether a person is resident in an institution. There are two codes to denote care-homes (‘93’ and ‘98’ for residential and nursing homes, respectively) [14]. The CHI Institution Flag field was extracted and classified as care-home (for codes 93 and 98) or not care-home as a binary allocation.
- Exact address matching: CHI address record exactly matches the concatenated address (using all available address fields) of a care-home in the Care Inspectorate list (binary allocation).
- Postcode matching: CHI address postcode exactly matches the postcode of a care-home address in the Care Inspectorate list after removing spaces (binary allocation). Missing postcodes in CHI was treated as ‘not a care-home address’ rather than excluded since routine data always contains records with missing postcode.
- Phonics matching: Metaphonics is a computational technique to convert a string variable, into phonetics based on their pronunciation in English [15]. These can be compared using SoundX in which words are given a numerical value and compared to other words which sound alike [15]. Metaphonics and SoundX match to an eligible care-home address in the Care Inspectorate list, including care-home postcode (creates a score from 0 [no match] to 100 [perfect match]) (Appendix 1, available at *Age and Ageing* online).
- Markov matching: A Markov model is a decision analysis tool which models possible outcomes and computes a probability based score as a result [16]. A Markov model using Scotland-wide data was generated to take a string of words and word pairs as predictors of a known result flag (the CHI Institution Flag plus the Health Board of residence since care-homes in different regions often have similar names). The Markov was used to match to an eligible care-home address in the Care Inspectorate list (creates a score from 0 [no match] to 100 [perfect match]) (Appendix 1, available at *Age and Ageing* online).

### Analysis

Index test assessments were compared to the reference standard. For the index tests with binary outcomes, sensitivity, specificity, positive predictive value (PPV) and NPV were calculated after creating two-by-two tables for each index test with exact 95% confidence intervals calculated [17]. There are no published cut-offs to define positivity for the two index tests with 0–100 scores (Phonics and Markov). Receiver Operating Characteristic (ROC) curves were therefore plotted, with area under the Receiver Operating Curve (AUROC) statistics with 95% Wald confidence limits calculated. Three methods were used to determine cut-offs: (i) a single cut-off for each test based on the clinical judgement of the research team which favoured maximising PPV (>90%) while preserving adequate sensitivity (>80% if possible, >90% ideally), (ii) the value at which sensitivity is equal to specificity and (iii) the Youden Index, which is the true positive rate minus the false positive rate [18, 19]. These approaches were selected after review of the published literature for situations where cut-off values have not been defined.

### Permissions

HIC Standard Operating Procedures have been reviewed and approved by the NHS East of Scotland Research Ethics Committee, and permission to analyse the data in the ISO27001 and Scottish Government accredited safe haven was obtained from the NHS Fife and NHS Tayside Caldicott Guardians. Analysis was carried out in SAS version 9.4.

## Results

### Participants

Of the 20,000 address records examined, 1,455 were considered by reference-standard classification to be ‘care-home addresses’ (7.3%). The proportion of care-home addresses varied across the four samples from 4.3 to 11.1%, primarily because of expected higher prevalence in the ≥65-year-olds versus the whole population sample (Table 1).

**Table 1.** Results comparing the performance of the binary methods in four samples of 5,000 addresses each from Fife and Tayside

	NHS Fife Population 213 (4.3%) true care-home addresses Estimate % (95% CI)	NHS Tayside Population 253 (5.1%) true care-home addresses Estimate % (95% CI)	NHS Fife ≥65-year-olds 556 (11.1%) true care-home addresses Estimate % (95% CI)	NHS Tayside ≥65-year-olds 431 (8.6%) true care-home addresses Estimate % (95% CI)
CHI Institution Flag	121 Identified as care-home	154 Identified as care-home	327 Identified as care-home	394 Identified as care-home
Sensitivity	55.8% (48.9–62.5)	59.7% (53.3–65.7)	58.6% (54.4–62.7)	89.3% (85.9–92.0)
Specificity	99.9% (99.8–99.9)	99.9% (99.7–99.9)	99.9% (99.8–99.9)	99.8% (99.6–99.9)
Positive predictive value	99.2% (94.8–99.9)	98.1% (93.9–99.5)	99.6% (98.0–99.9)	97.7% (95.6–98.9)
Negative Predictive value	98.1% (97.6–98.4)	97.9% (97.4–98.3)	95.1% (94.4–95.7)	99.0% (98.7–99.3)
Exact address match	0 Identified as care-home	0 Identified as care-home	0 Identified as care-home	0 Identified as care-home
Sensitivity	0.0% (0.0–2.2)	0.0% (0.0–1.9)	0.0% (0.0–0.1)	0.0% (0.0–1.1)
Specificity	100.0% (99.9–100.0)	100% (99.9–100.0)	100% (99.9–100.0)	100% (99.9–100)
Positive predictive value	–	–	–	–
Negative Predictive value	95.7% (95.1–96.3)	94.9% (94.3–95.5)	88.9% (87.9–89.7)	91.4% (90.5–92.1)
Postcode match	251 Identified as care-home	252 Identified as care-home	580 Identified as care-home	454 Identified as care-home
Sensitivity	90.2% (85.3–93.7)	78.2% (72.6–83.1)	89.2% (86.3–91.6)	89.6% (78.6–94.4)
Specificity	98.8% (98.4–99.1)	98.9% (98.5–99.1)	98.1% (97.7–98.5)	98.5% (98.1–98.8)
Positive predictive value	77.3% (71.5–82.2)	78.6% (72.9–83.4)	85.5% (82.3–88.2)	85.0% (81.3–88.1)
Negative Predictive value	99.6% (99.3–99.7)	98.8% (98.5–99.1)	98.6% (98.2–98.9)	99.0% (98.7–99.3)

CHI, Community Health Index; Negative predictive value—the proportion of addresses identified as non-care-home addresses which are not care-homes; Positive predictive value—the proportion of addresses identified as care-homes which are care-home addresses; Sensitivity—the proportion of care-home addresses correctly identified by each method; Specificity—the proportion of non-care-home addresses correctly identified by each method.

### Test results

Each of the index tests are considered in turn, with results reported for the four samples (Tables 1 and 2; Supplementary Figure 1, available at *Age and Ageing* online). Appendix 2, available at *Age and Ageing* online shows the STARD flow diagram for each index test. Specificity and negative predictive value (NPV) were consistently high across all methods, whereas there was more variation in sensitivity and PPV which were inversely related. CHI Institution Flag sensitivity varied across samples ranging from 55.8 to 89.3%. It had a consistently excellent PPV (97.7–99.6%). Exact address matching failed for all 20,000 addresses examined. Postcode matching achieved sensitivity 78.2–90.2%, but the PPV was poorer (77.3–85.5%), particularly among the whole population samples.

**Table 2.** Results comparing the performance of the cut-off methods in four samples of 5,000 addresses each from Fife and Tayside

	NHS Fife Population 213 (4.3%) true care-home addresses Estimate (95% CI)	NHS Tayside Population 253 (5.1%) true care-home addresses Estimate (95% CI)	NHS Fife ≥65-year olds 556 (11.1%) true care-home addresses Estimate (95% CI)	NHS Tayside ≥65-year olds 431 (8.6%) true care-home addresses Estimate (95% CI)
Phonics score area under the curve	0.950 (0.930–0.970)	0.863 (0.835–0.890)	0.924 (0.909–0.939)	0.934 (0.918–0.950)
Markov score area under the curve	0.957 (0.937–0.976)	0.935 (0.914–0.956)	0.966 (0.956–0.977)	0.986 (0.979–0.994)
Phonics score researcher-defined cut-off	200 (cut-off ≥13)	199 (cut-off ≥13)	485 (cut-off ≥13)	400 (cut-off ≥13)
Sensitivity	87.4% (82.1–91.4)	72.7% (66.7–78.0)	84.9% (81.5–87.7)	87.0% (83.4–89.9)
Specificity	99.7% (99.5–99.8)	99.6% (99.4–99.8)	99.7% (99.4–99.8)	99.5% (99.2–99.6)
Positive predictive value	94.0% (89.5–96.7)	92.4% (87.6–95.5)	97.3% (95.3–98.5)	93.8% (90.8–95.8)
Negative Predictive value	99.4% (99.2–99.6)	98.5% (98.1–98.8)	98.1% (97.6–98.5)	98.7% (98.4–99.1)
Phonics score maximising Youden’s J	222 (cut-off ≥0.50)	199 (cut-off ≥13.0)	485 (cut-off ≥12.9)	400 (cut-off ≥13.0)
Sensitivity	90.2% (85.3–93.7)	72.7% (66.7–78.0)	84.9% (81.6–87.7)	87.0% (83.4–90.0)
Specificity	99.4% (99.1–99.6)	99.7% (99.5–99.8)	99.7% (99.5–99.8)	99.5% (99.2–99.6)
Positive predictive value	87.4% (82.1–91.3)	92.5% (87.6–95.6)	97.3% (95.3–98.5)	93.8% (90.8–95.8)
Negative predictive value	99.6% (99.3–99.7)	98.6% (98.2–98.9)	98.1% (97.7–98.5)	98.8% (98.4–99.1)
Markov score researcher-defined cut-off	201 (cut-off ≥29.6)	194 (cut-off ≥29.6)	501 (cut-off ≥29.6)	418 (cut-off ≥29.6)
Sensitivity	84.2% (78.5–88.7)	69.2% (63.0–74.7)	85.4% (82.2–88.2)	90.3% (86.9–92.8)
Specificity	99.6% (99.3–99.7)	99.6% (99.4–99.8)	99.4% (99.1–99.6)	99.4% (99.1–99.6)
Positive predictive value	90.0% (84.8–93.7)	90.2% (84.9–93.8)	94.8% (92.3–96.5)	93.1% (90.1–95.2)
Negative Predictive value	99.3% (98.9–99.5)	98.4% (97.9–98.7)	98.2% (97.8–98.6)	99.1% (98.8–99.3)
Markov score maximising Youden’s J	301 (cut-off ≥5.9)	399 (cut-off ≥5.0)	620 (cut-off ≥5.9)	589 (cut-off ≥4.9)
Sensitivity	90.7% (85.8–94.1)	85.8% (80.7–89.7)	92.6% (90.0–94.6)	97.2% (95.1–98.5)
Specificity	97.8% (97.3–98.2)	96.2% (95.6–96.7)	97.6% (97.1–98.1)	96.3% (95.7–96.8)
Positive predictive value	64.8% (59.1–70.1)	54.4% (49.4–59.3)	83.1% (79.8–85.9)	71.1% (67.3–74.7)
Negative predictive value	99.6% (99.3–99.7)	99.2% (98.9–99.4)	99.1% (98.7–99.3)	99.7% (99.5–99.9)
Markov score sensitivity = specificity	555 (cut-off ≥1.4)	750 (cut-off ≥1.6)	816 (cut-off ≥2.2)	572 (cut-off ≥6.1)
Sensitivity	92.6% (88.0–95.5)	88.9% (84.2–92.4)	93.9% (91.5–95.7)	96.5% (94.2–97.9)
Specificity	92.6% (91.8–93.3)	88.9% (88.0–89.8)	93.4% (92.6–94.1)	96.6% (96.0–97.1)
Positive predictive value	35.9 (31.9–40.0)	30.0% (0.27–0.33)	63.9% (60.6–67.3)	72.7% (68.8–76.3)
Negative predictive value	99.6 (99.4–99.8)	99.3% (99.0–99.6)	99.2% (98.9–99.4)	99.7% (99.4–99.8)

Number in bold is the number the method identified as a care-home using the cut-off value quoted. Area under curve—a measure of evaluating how well each test discriminates between care-home and non-care-home addresses. The closer the value is to 1, the better the performance of the test. Cut-offs are researcher-defined aiming to maximise positive predictive value (ideally >95%) with adequate sensitivity (ideally >80%), Youden’s J (sensitivity plus specificity minus one with values closer to one indicating better test performance), and sensitivity = specificity (which did not exist for Phonics so not shown). Sensitivity—the proportion of care-home addresses correctly identified by each method; Specificity—the proportion of non-care-home addresses correctly identified by each method; Negative predictive value—the proportion of addresses identified as non-care-home addresses which are not care-homes; Positive predictive value—the proportion of addresses identified as care-homes which are care-home addresses.

Phonics scores were distributed across a relatively small number of values (14 values, most commonly zero) (Supplementary Figure 1, available at *Age and Ageing* online). AUROC statistics varied from good to excellent (0.86–0.95). Using a researcher determined cut-off of ≥13 resulted in PPV of 92.4–97.3% and sensitivity of 72.7–87.4% (Table 2). Results using Youden’s Index to determine the cut-off were similar (Table 2). It was not possible to identify a cut-off at which sensitivity was equal to specificity due to the limited range of values.

The Markov model had excellent performance with AUC values between 0.93 and 0.98 (Supplementary Figure 2 and Table 2, available at *Age and Ageing* online). A researcher selected cut-off of ≥29.6 is presented for all four samples, ensuring PPV of 90.0–94.8%. This resulted in sensitivity of 69.2% in the Tayside population sample and sensitivity between 84.2% and 90.3% in the other three samples. Using Youden’s Index, cut-offs between ≥4.9 and ≥5.9 achieved sensitivity 85.8–97.2%, but the associated PPVs ranged from 54.4% to 83.1% (Table 2). The cut-off values to define test positivity varied between ≥1.4 and ≥6.1 to achieve the point at which sensitivity was equal to specificity (Table 2).

All methods apart from exact address matching performed better in the over-65 population than the whole population samples. The CHI Institution Flag had a sensitivity of 55.8% and 59.7% in the population samples, compared to 58.6% and 89.3% in ≥65-year olds sample. Postcode matching had an improved PPV in the ≥65-year olds sample of 85.0% and 85.5% compared to 77.3% and 78.6% in the population samples. Similarly, the Phonics and Markov models achieved higher PPVs of 93.8–97.3% and 93.1–94.8%, respectively, in the older adult samples, compared to 92.4–94.0% and 90.0–90.2% in the whole population samples. Comparing the two health board areas, the CHI Institution Flag was more sensitive in NHS Tayside compared to NHS Fife, particularly among the ≥65-year olds sample (89.3% vs. 58.6%).

## Discussion

### Statement of principal findings

All the measures examined have a consistently excellent specificity and NPV. The existing NHS Scotland CHI Institution Flag has a very high PPV but less good sensitivity. Put another way, where it identifies an address as being a care-home then it is almost always correct, but it fails to identify 11–45% of care-home resident addresses depending on the sample. Exact address matching comprehensively failed. Postcode matching was more sensitive than the existing CHI Institution Flag but at the cost of misclassifying more private addresses as being care-homes. Both the Phonics and Markov methods had better sensitivity than the CHI Institution Flag and reasonable PPV. The choice of cut-off to define test positivity affects the balance between sensitivity and PPV, but all cut-offs performed well. The differences in performance between the whole population and ≥65-year old samples and those seen in the two health board areas are important to explore. These suggest underlying differences in the recording of address information and coding practices of the institution flag. There may also be spectrum bias [20] related to the performance of the methods based on the different case mix between the older adult and whole population samples.

### Strengths and weaknesses of the study

A key strength is that the reference standard was robustly created for 20,000 addresses independently of the index test calculations. The Phonics and Markov methods are innovative and represent techniques which could be used to identify care-home residents in routine data, with the choice informed by the needs of the research question. Phonics matching can be applied to any set of paired patient and care-home addresses. The Markov was trained using the CHI database so may not generalise outside Scotland, but the same approach can be used in other contexts where there is some kind of flag available for training, including flags created by initial manual classification. Both methods outperformed the CHI Institution Flag, indicating a role for informatics to better identify care-home residents from their routinely recorded address. The work is limited in that it only examined two health board areas of Scotland. Given the variations in data quality identified, the results may not be generalizable across the other health board areas and formal evaluation would be useful. The prevalence of care-home addresses in the samples are higher than the prevalence of care-home residents in the population; this is likely to affect the generalisability of the estimates of test accuracy presented [21]. The higher prevalence is likely to be partly explained by the analysis being based on CHI address data entries, rather than individual living residents. However, our manual classification of addresses identified widespread use of historic care-home names (i.e. services which had been cancelled by the Care Inspectorate, but where an alternative care-home service was present on that site). This may indicate the presence of live CHI records for patients who have moved or died, consistent with the higher than expected prevalence of care-home addresses in the test samples. All these problems are likely to be common in other contexts, and future external validation using routinely-collected address data in other areas is therefore required.

### Strengths and weaknesses in relation to other studies

At present, the CHI Institution Flag and postcode matching are the only methods available for researchers in Scotland to identify care-home residents using CHI records. For people who are admitted to hospital, routine coding of ‘admission from’ and ‘discharge to’ variables can record care-home residence, but this is often incorrect [22]. The method developed by the Nuffield Trust uses postcode matching combined with individual age ≥75 years, with a reported PPV of 87% [6]. Researchers in the East Midlands of England, have tested an algorithm to match addresses from hospital admission data, achieving a PPV of 100% [23]. However, the external validation of their algorithm lacked an independent gold standard reference meaning that its performance is not known for certain [24], and any method solely based on hospital admission data will not identify all care-home residents. This is a developing area of interest and it is essential proposed methods are described and evaluated to understand their strengths and limitations.

### Meaning of the study

Identifying care-home residents using address-level data remains challenging and it is clear that no method examined here optimises *both* sensitivity and PPVs to ideal levels (>95%). In practice therefore, researchers and policy-makers will have to trade off feasibility alongside which parameter they wish to optimise when selecting a method. Maximising the PPV will ensure that those identified as care-home residents are more likely to actually be in a care-home, whereas maximising sensitivity will ensure more care-home residents are included. For example, researchers wishing to study care-home residents as a sub-group within the population with respect to prescribing, may be happy to accept a poorer sensitivity to ensure high PPV since false negatives (care-home residents misclassified as living in their own home) will be a small proportion of the comparison population. This will produce less biased estimates than if a significant proportion of those classified as care-home residents are false positives. Alternatively, if researchers intend to manually classify addresses, then using Phonics or Markov with a cut-off that maximises sensitivity would markedly reduce the number of addresses needing manual classification allowing more efficient creation of a reference-standard dataset, although that this will not be an absolutely complete population sample.

### Unanswered questions and future research

All methods had somewhat varying performance depending on the dataset used, with better performance in over-65 populations and differences between the two Health Boards particularly for the CHI Institution Flag already used by NHS Scotland. The CHI flag is often the only marker which researchers have to identify care-home residents in Scotland, and our findings indicate use of this measure will miss a large minority of care-home residents in both health boards, but more in Fife than Tayside. This finding requires further exploratory work in other areas and collaboration with NHS Scotland’s Practitioner Services Division to improve the data quality informed by our findings. More generally, the methods described have potential application in other countries which use address-based datasets to classify care-home residency. The Phonics method can be applied to any text data. The Markov method requires a gold standard to be trained on, which would have to be created manually if no suitable variable exists. All methods require external validation.

A key challenge remains for practitioners and researcher in how to accurately identify those whose stay in care-homes is temporary, e.g. for respite or intermediate care and those who are newly admitted to a care-home following a hospital admission. This requires responsive Information Technology systems whose data can be contemporaneously and accurately updated. Such populations are of specific interest in evaluating services and innovations and this is therefore a priority area to improve data quality.

In conclusion, this study shows that automated methods for address matching have excellent but not ideal performance in identifying addresses which are care-homes. Improving the reliable identification of care-home residents in routine data is the first step in improving representation of this vulnerable and complex population in service evaluation, research and evidence-based policy making.

**Key points**

- Current health data systems do not enable reliable identification of care-home residency.
- This represents a significant limitation for data-driven, inclusive research and service improvement.
- Existing and novel Scottish methods for identifying residency have been examined.
- Approaches adopted will vary depending on the requirement for greater precision or inclusivity.
- Opportunities identified should inform improvement of systems of health data collection.

## Supplementary Material

Supplementary Data
