# Supplementary material for: Identifying care-home residents in routine healthcare datasets: a diagnostic test accuracy study of five methods
Source: Age Ageing. 2018 Jul 26;48(1):114–21. doi: 10.1093/ageing/afy137 (PMC6322499; doi:10.1093/ageing/afy137)

### Supplementary Materials

Supplementary Figure 1: Receiver Operating Characteristic Curve using Phonics Method


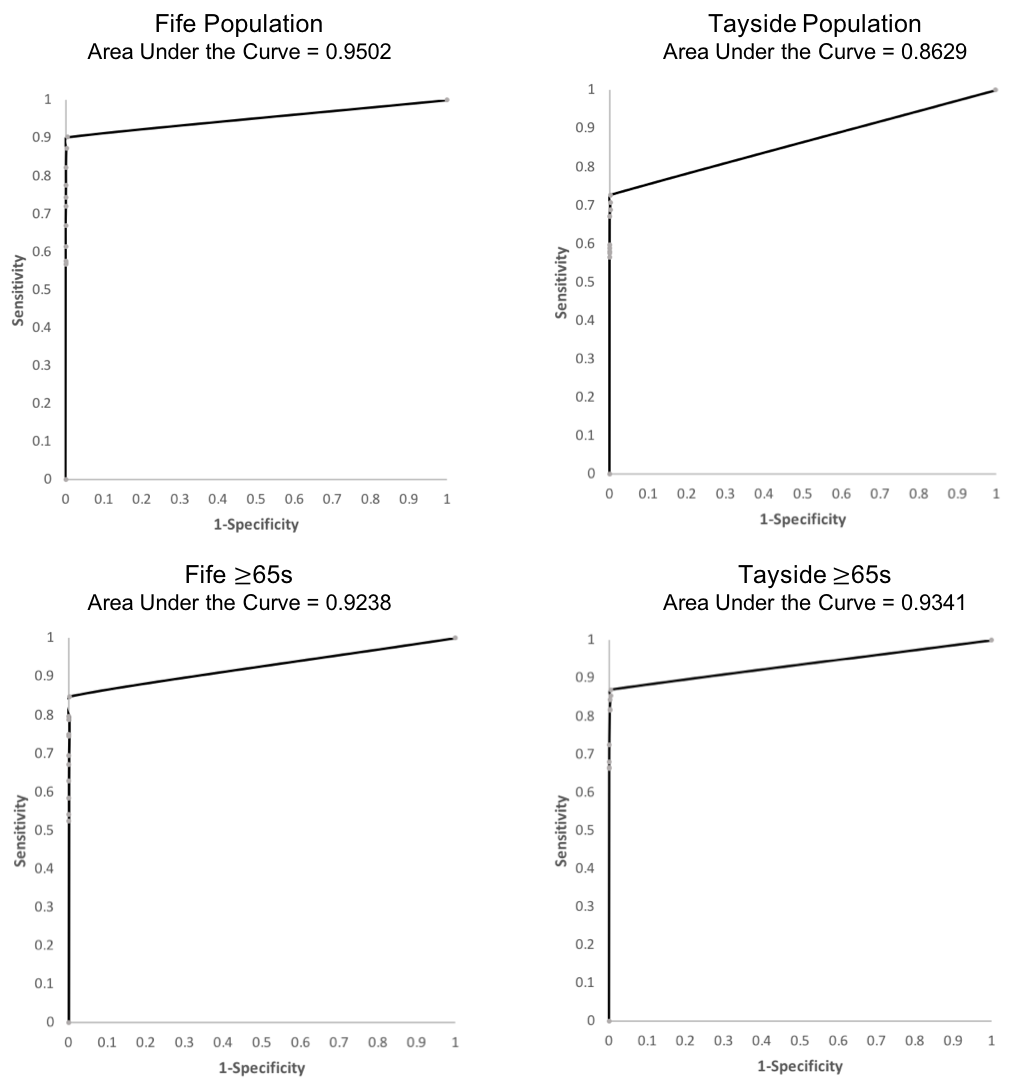


Supplementary Figure 2: Receiver Operating Characteristic Curve using Markov Method


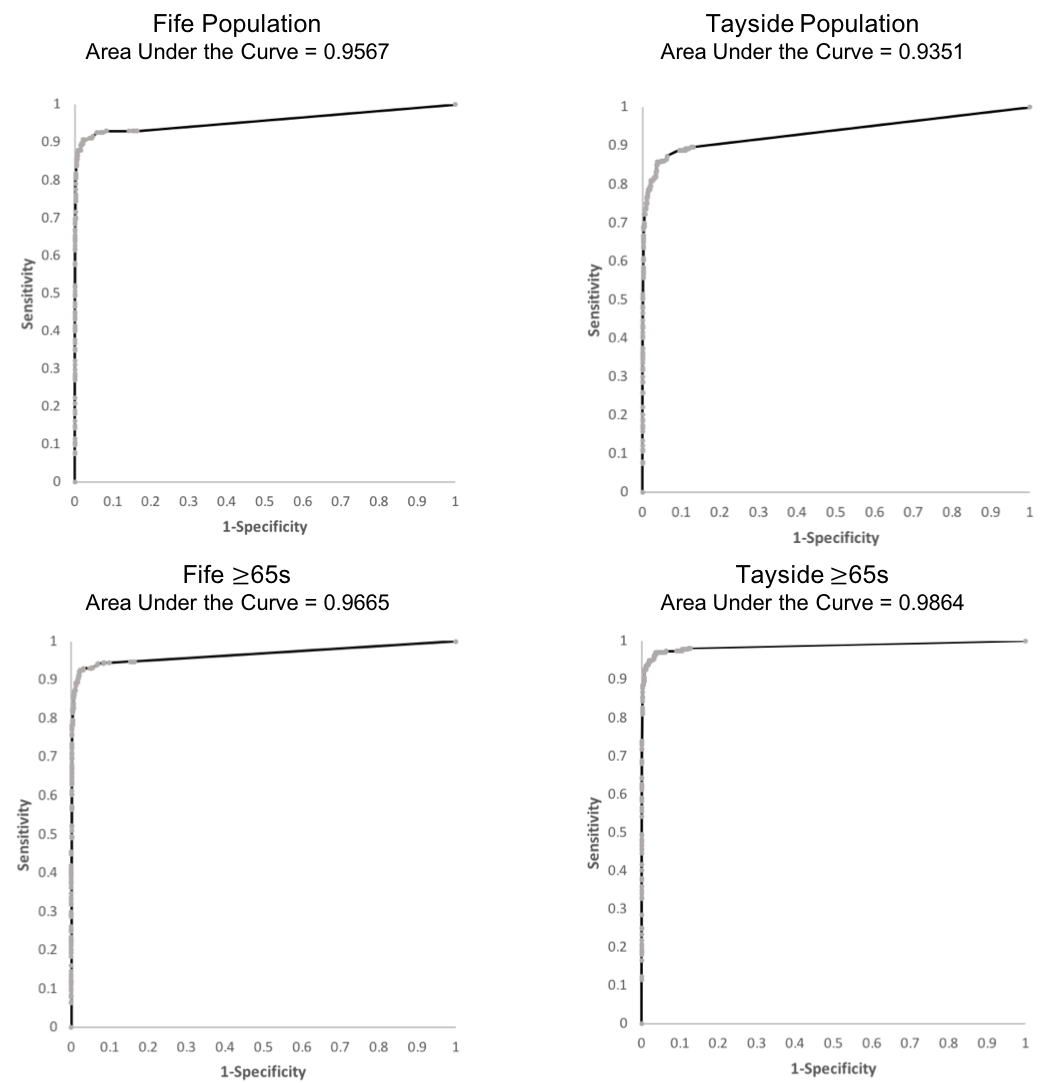


### Appendix 1 – Phonics and Markov model methods

### Phonics

Metaphonics is a computational technique to convert each word of a string variable, such as an address, into phonetics (metaphonic words), based on how they are pronounced in English [20]. These can then be compared using a second approach, SoundX, in which words are given a numerical value and compared to other words which sound alike [20]. A match score % is calculable for the original string variable and the address it has been matched to, based on their similarity.

The text below provides a detailed description of how to operationalise the Phonics model used in this case.

#### Algorithm (Where A denotes Care Inspectorate List and B denotes CHI record):

Input record

Concatenate all lines in record (A)

For each record in patient addresses (B)

If A.Postcode does not match B.Postcode - **no match**

Else Extract all numbers in A and B and concatenate to A.AllNums and B.AllNums

If A.AllNums matches B.AllNums – **100% match**

Else For each patient address (B)

Get all care-home addresses A that has a matching postcode with B

If no B.Postcode match any A.Postcode – **no match**

If B.Postcode matches A.PostCode

Save all records in A that has a matching postcode with B

Concatenate B.Address1, B.Address2, B.Address3 and B.Address4 🡪 B.Address

Convert all words in B.Address to Metaphonic 🡪 B.Metaphonic

For each Matching address A:

Concatenate A.Address1, A.Address2, A.Address3 and A.Address4 🡪 A.Address

Convert A.Address to Metaphonic 🡪 A.Metaphonic

For each word in B.Metaphonic:

Get the Soundex Score comparing with each word in A.Metaphonic

If there is no exact match (Soundex score not equal to 4)

Save the highest score only for each word.

If a word got a matching score of 0 (does not match any word in the care-home address reduce the total result of the whole B.Metaphonic by 3

Divide the total score of the whole address (B.Metaphonic) by number of word and then by 4 (to get the average for score and number of words) then multiply the result by 100.

Get the highest average score for B.Address after comparing with all matching A.Address set (based on Postcode) as final Phonics score for B.Address and save the ID of the matching A.Address with the highest average score.

Using the above algorithm each patient address was compared against a matching care-home address and a matching score (Phonics score) was recorded.

#### Markov development

The Markov was developed using input variable information from the national CHI dataset and the Care Inspectorate list of services.

## CHI dataset

| AddressLine1 | AddressLine2 | AddressLine3 | AddressLine4 | Postcode | Date | CHIInstitutionFlag | Healthboard |
| --- | --- | --- | --- | --- | --- | --- | --- |

## Care Inspectorate list

| Care home type | Care home subtype | Name | AddressLine1 | AddressLine2 | AddressLine3 | Postcode |
| --- | --- | --- | --- | --- | --- | --- |

The model was generated to take a string of word tokens and score word pairs as predictors of a known result flag. This required creation of a database table which extracted AddressLine1 and AddressLine2 as ComboAddress, CHIInstitutionFlag where positive for residential or nursing home to create IsCareHome field and extracted Healthboard.

## Database Table


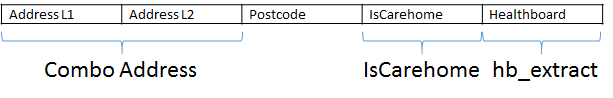


A query was written to select the columns you wish to extract data from as a field called ComboAddress. It is also necessary to select the IsCarehome (1/0) field and hb_extract as your healthboard field, for the purposes of these analyses confided to T (Tayside) and F (Fife).

Take each string and split it into a word string. Take each pair in the word string. If the pair was seen before increment the count positive/negative. If it hasn’t been seen before record the pair. The resulting output table can be used to classify new cases. Then take the total weight and divide it by the number of matches (1 + 0.5 / 2) = 75% match. The patient address in the CHI record is divided into five parts (Address line 1-4) plus the postcode. The postcode is ignored as Markov is concerned only with the free text parts. Early testing of the Markov process revealed that using different parts of the patient address could result in different Markov scores. In order to optimize the Markov performance, the score of the whole address and the first and second line of each address were tested individually, these three Markov values were compared and the highest value was recorded as the Markov value for the address.

The first model was trained on a random sample of 1,000,000 CHI records from all health boards in Scotland. The address in the patient record was marked as a care-home if the ISD Institution Flag was either 93 or 98, anything else was considered as not a care-home address. The model was retrained using the same data set from all over Scotland, for individuals who were living in these addresses on 01^st^ March 2017 to make sure that the addresses were current and that the Institution flag was as complete as possible.

**References**

1. Beider A, Morse SP. Phonetic Matching: A Better Soundex. Association of Professional Genealogists Quarterly [serial on the Internet]. 2010 6th May 2017 [cited 2017 6th May]; 25: Available from: <http://stevemorse.org/phonetics/bmpm2.htm>

2. York Health Economics Consortium. Markov Model. York 2016 [cited 2017 25th September]; Available from: <http://www.yhec.co.uk/glossary/markov-model/>.

### Appendix 2 – STARD flow diagrams for each index test

In all cases, all 20,000 addresses are examined with all five index tests and the reference standard.

#### 1. CHI Institution Flag


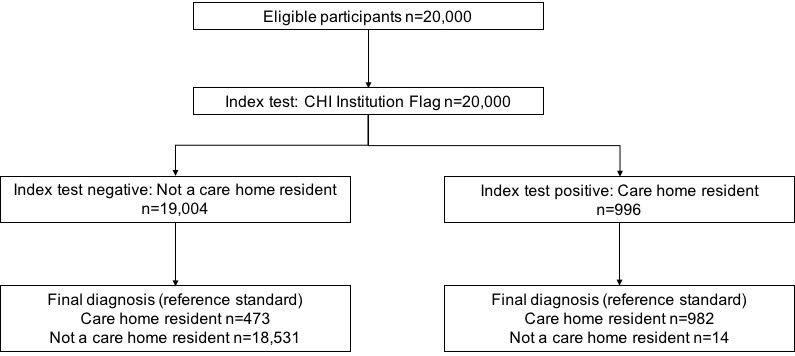


#### 2. Exact address match


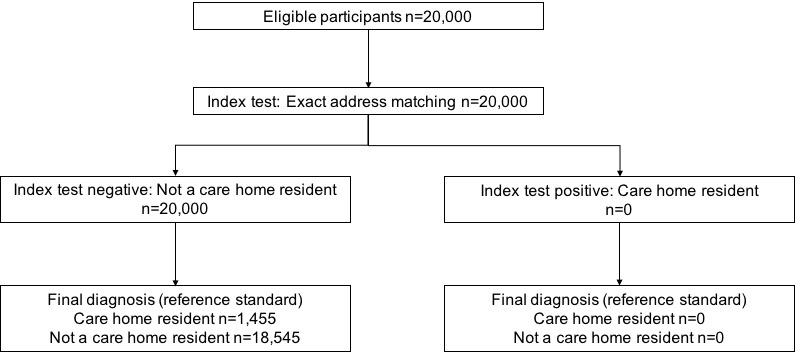


#### 3. Postcode Match


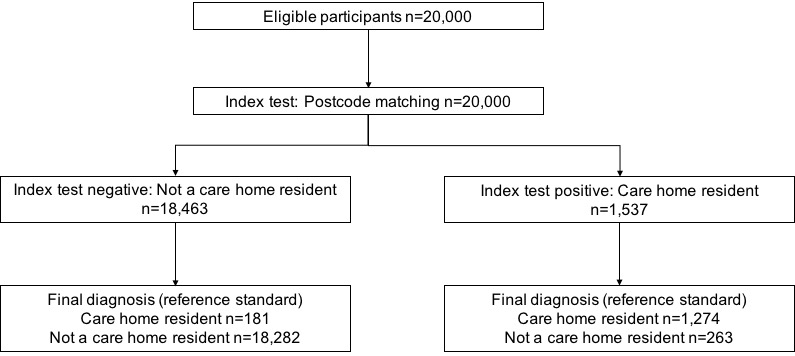


#### 4. Phonics matching (cut-off ≥13.0)


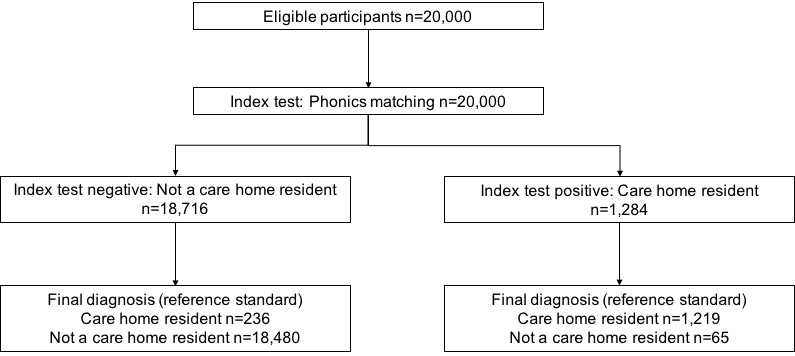


#### 5. Markov matching (cut-off ≥29.6)


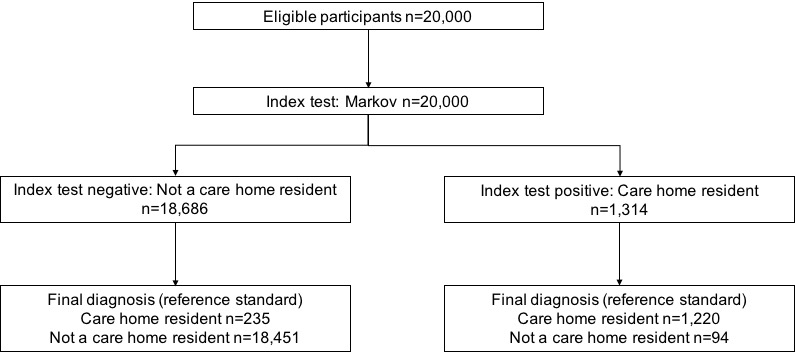

Supplement: Supplementary Data [file afy137_aa-18-0226-file003.docx]
